# Diagnostic Accuracy and Prognostic Value of Flow Cytometry Versus PCR-Based Minimal Residual Disease Assessment in Leukemia: A Systematic Review and Meta Analysis

**DOI:** 10.7759/cureus.113198

**Published:** 2026-07-22

**Authors:** Afaf Alalwy, Bandar S Alshreef, Iman Semoud, Bayan Ahmed, Samaher A Alyousfi, Manal M Sayed, Tahani R Alshammari, Tahani A Alaslani, Abdulrhaman F Alanazi, Shatha A Alaufi, Ali S Metwaly

**Affiliations:** 1 Forensic Toxicology and Criminal Evidence, University of Tabuk, Tabuk, SAU; 2 Healthcare Management, College of Applied Medical Sciences, Shaqra University, Shaqra, SAU; 3 Hematology, University of Algiers Benyoucef Benkhedda, Algiers, DZA; 4 Hematology, Saudi Arabia Ministry of Health, Jazan, SAU; 5 Laboratory Medicine, Al Borg Diagnostics, Abha, SAU; 6 Clinical Pathology, Thagher Hospital, Jeddah, SAU; 7 Laboratory Medicine, Hail University, Hail, SAU; 8 Hematology Laboratory, King Fahad Hospital Jeddah, Jeddah, SAU; 9 Clinical Laboratory Science, SDMC Diriyah Health-Medical Complex, Riyadh, SAU; 10 General Medical Laboratory, Alborg Laboratories, Madinah, SAU; 11 Precision Medicine, Faculty of Pharmacy, Alexandria University, Alexandria, EGY

**Keywords:** diagnostic accuracy, flow cytometry, leukemia, meta-analysis, minimal residual disease, polymerase chain reaction, prognostic value

## Abstract

Measurable/minimal residual disease (MRD) is the most critical prognostic biomarker in leukemia. Multiparameter flow cytometry (MFC) and polymerase chain reaction (PCR)-based assays are the clinical standards for MRD detection; however, inter-method discordance complicates the clinical decision-making process. A systematic review and meta-analysis were conducted to evaluate the diagnostic accuracy of MFC compared to PCR and to delineate the prognostic significance of discordant MRD results. Following the Preferred Reporting Items for Systematic Reviews and Meta Analyses (PRISMA) guidelines, major databases were systematically searched for studies comparing MFC and PCR-based MRD assessments in acute lymphoblastic leukemia (ALL) and acute myeloid leukemia (AML). Diagnostic test accuracy was pooled using a bivariate random-effects model, and prognostic hazard ratios were synthesized using the restricted maximum likelihood models. Twenty-three non-randomized studies met the inclusion criteria of this review. In the diagnostic meta-analysis (12 studies), MFC demonstrated a pooled sensitivity of 76.2% (95% CI, 55.2-89.3) and a high pooled specificity of 96.0% (95% CI, 93.2-97.7) when compared with PCR, yielding an excellent area under the curve (AUC) of 0.965. Meta-regression identified leukemia subtype as a significant moderator (*p*=0.007), with MFC exhibiting lower diagnostic odds ratios in AML than in ALL. Survival analysis of MFC/PCR discordance (six studies) yielded a non-significant pooled HR of 1.27 (95% CI, 0.34-4.70) with extreme heterogeneity (*I*^2^=89.2%), driven by the comparative baseline: isolated PCR positivity (MFC−/PCR+) conferred a significant survival advantage when compared to double-positive disease, but portended a significantly higher relapse risk when compared to double-negative disease. MFC is a highly specific and rapid modality for MRD detection, but it lacks the analytical sensitivity of PCR, particularly in AML. MFC/PCR discordance does not represent a uniform biological risk state; rather, its prognostic implications are highly context-dependent. The integration of both modalities provides a synergistic approach to optimize risk stratification and therapeutic tailoring in precision hematology.

## Introduction and background

Leukemia encompasses a heterogeneous group of hematologic malignancies characterized by uncontrolled proliferation of abnormal hematopoietic progenitors. Despite the high morphological complete remission rates achieved following standard induction chemotherapy, disease relapse is the primary cause of treatment failure and mortality in both acute lymphoblastic leukemia (ALL) and acute myeloid leukemia (AML) [1,2]. The detection of measurable/minimal residual disease (MRD), defined as the persistence of submicroscopic levels of leukemic cells below the threshold of conventional morphological assessment, has shifted the clinical paradigm in onco-hematology. MRD is established as the most powerful, independent prognostic biomarker for predicting relapse-free and overall survival [1,2], and its robust predictive value has led to its integration into routine clinical practice for risk stratification, guiding crucial therapeutic decisions such as the (de)-intensification of chemotherapy, selection of conditioning regimens for allogeneic hematopoietic stem cell transplantation (HSCT), and deployment of targeted immunotherapies [2,3].

Multiparameter flow cytometry (MFC) and polymerase chain reaction (PCR)-based assays are the principal modalities for MRD monitoring [1]. MFC identifies residual leukemic blasts by detecting leukemia-associated immunophenotypes (LAIPs) or different-from-normal (DfN) antigenic profiles [4,5]. The primary advantages of MFC lie in its broad applicability, being useful in over 90% of patients regardless of their underlying molecular subtype, along with its rapid turnaround time and capacity to assess cell viability [1,6]. However, MFC is limited by a lower analytical sensitivity (10-4 to 10-5), the necessity for fresh marrow samples, and susceptibility to both false-negative results driven by immunophenotypic shifts and false-positive results stemming from regenerating normal hematopoietic progenitors [5,7].

PCR-based techniques, including real-time quantitative PCR (RQ-PCR) and reverse-transcription quantitative PCR (RT-qPCR), offer superior analytical sensitivity, detecting one leukemic cell in 105 to 106 normal cells [1,2]. In ALL, PCR targets patient-specific clonal immunoglobulin (Ig) and T-cell receptor (TCR) gene rearrangements, whereas in AML, it targets recurrent genetic fusions (e.g. RUNX1::RUNX1T1, CBFB::MYH11) or specific mutations (e.g. NPM1 or WT1 overexpression) [1,4,6]. Despite its high sensitivity and rigorous international standardization, PCR-based MRD assessment is time-consuming, expensive, and limited in its applicability. Suitable molecular targets are absent in approximately 50% of AML cases, and Ig/TCR monitoring in ALL requires labour-intensive development of patient-specific primers [1,2]. Furthermore, the clinical specificity of PCR can be confounded by the amplification of DNA from non-viable apoptotic cells post-treatment or the harmless persistence of pre-leukemic clones (e.g. clonal hematopoiesis) that lack immediate relapse potential [2,8].

Given their distinct biological targets and methodological thresholds, MFC and PCR yield discordant results in clinical settings. The concordance between the two assays is variable and often time-point-dependent, influenced by the leukemia subtype, specific molecular targets, and phase of treatment [7,8]. For instance, isolated PCR positivity (MFC-/PCR+) may reflect the superior analytical sensitivity of molecular techniques or the persistence of non-clonogenic transcripts without clinical relevance [3,8], whereas isolated MFC positivity (MFC+/PCR-) may arise from the loss of molecular targets due to clonal evolution or limitations in PCR primer design [3,4,8]. Previous studies have demonstrated that integrating both methods can refine prognostic stratification; for example, combining MFC with WT1 or NPM1 PCR assessment has been shown to more accurately predict post-transplant relapse in AML [3,4]. However, the precise clinical and prognostic significance of these discordant states remains a subject of debate, creating challenges for clinicians attempting to tailor interventions [3,7].

Although numerous primary studies have investigated the use of MFC and PCR in MRD assessment, evidence remains fragmented across disparate clinical settings, pediatric and adult cohorts, and varying therapeutic time points. There is a need to synthesize this literature to determine which modality or combination thereof provides superior diagnostic performance and prognostic information. Therefore, this systematic review and meta-analysis aimed to evaluate and compare the diagnostic accuracy and prognostic value of MFC versus PCR-based MRD assessment in patients with leukemia. This study seeks to inform evidence-based clinical decision-making, optimize MRD-guided management strategies, and highlight areas requiring methodological harmonization in precision hematologic oncology by clarifying the clinical impact of method-specific MRD detection and exploring the implications of inter-method discrepancies.

## Review

Methods

Protocol Registration and Reporting Guidelines

This systematic review and meta-analysis was conducted in accordance with the Preferred Reporting Items for Systematic Reviews and Meta-Analyses (PRISMA) 2020 guidelines [9]. The review protocol was prospectively registered in the International Prospective Register of Systematic Reviews (PROSPERO; CRD420261359922) before the initiation of the literature screening.

Search Strategy and Information Sources

A systematic search of the literature was performed across major electronic databases, including PubMed/MEDLINE, Embase, Cochrane Central Register of Controlled Trials (CENTRAL), Scopus, Web of Science, and Science Citation Index Expanded (SCIE). The grey literature was searched to mitigate publication bias. The search strategy utilized a combination of controlled vocabulary (e.g. medical subject headings (MeSH) and Emtree terms) and free-text keywords constructed using Boolean operators (AND, OR, NOT) and truncations. The key search terms included "Leukemia", "Minimal Residual Disease", "Measurable Residual Disease", "Flow Cytometry", "Polymerase Chain Reaction", "Diagnostic Accuracy", and "Prognosis". No language or date restrictions were imposed. Furthermore, forward citation searching (snowballing) and backward reference list checking of the included studies and relevant reviews were performed to identify additional eligible literature.

Eligibility Criteria

The study inclusion was determined using a predefined PICOS (Population, Intervention/Index test, comparator/reference standard, Outcomes, Study design) framework. The population was patients of any age (pediatric or adult) with a confirmed diagnosis of leukemia, including acute ALL, AML, chronic lymphocytic leukemia (CLL), and chronic myeloid leukemia (CML). The intervention/index test was MRD assessment performed using MFC, and the comparator/reference standard was MRD assessment performed using PCR-based methodologies (e.g. RQ-PCR, RT-qPCR, allele-specific oligonucleotide PCR).

For diagnostic test accuracy (DTA), outcomes included sensitivity, specificity, positive/negative predictive values (PPV/NPV), diagnostic odds ratio (DOR), and area under the curve (AUC). For prognostic value, outcomes included overall survival (OS), event-free survival (EFS), progression-free survival (PFS), disease-free survival (DFS), and cumulative incidence of relapse. Non-randomized study types, including prospective and retrospective cohorts, longitudinal observational studies, and diagnostic accuracy studies, were included. We excluded case reports, series with <10 participants, animal studies, and reviews.

Study Selection and Data Extraction

Following the removal of duplicates, two investigators independently screened the titles and abstracts for eligibility. Full-text articles of potentially eligible studies were then independently evaluated, and disagreements were resolved by adjudication by a third reviewer. The inter-rater reliability for study inclusion was quantified using Cohen’s Kappa coefficient (κ) [10].

Data were independently extracted using a standardized pilot-tested extraction form. The extracted variables included study and patient characteristics, leukemia subtype, MRD methodologies, specific targets, timing of assessment, MRD thresholds, 2×2 contingency tables (true positive, false positive, false negative, true negative), and survival data (hazard ratios (HRs) and 95% confidence intervals (CIs)). If HRs were not explicitly reported, they were estimated from Kaplan-Meier survival curves using the Tierney method [11].

Quality Assessment and Risk of Bias

The methodological quality and risk of bias (RoB) of the included studies were appraised by two independent reviewers using tools specific to the study design. For diagnostic accuracy outcomes, the Quality Assessment of Diagnostic Accuracy Studies 2 (QUADAS-2) tool was used to evaluate bias across four domains: patient selection, index test, reference standard, and flow and timing [12].

Statistical Analysis and Data Synthesis

All statistical analyses were conducted using R software version 4.6.0 (R Foundation for Statistical Computing, Vienna, Austria), utilizing the meta, metafor, and mada packages [13].

*Diagnostic accuracy meta-analysis*: To account for the intrinsic correlation between sensitivity and specificity across varying study thresholds, a bivariate random-effects model was employed to calculate the pooled sensitivities, specificities, positive likelihood ratios (PLR), negative likelihood ratios (NLR), and DORs [14]. A hierarchical summary receiver operating characteristic (HSROC) curve was generated to visualize the overall diagnostic performance and calculate the AUC [15].

*Prognostic value meta-analysis*: For time-to-event outcomes (OS, EFS, DFS, and PFS), HRs and their corresponding standard errors were natural log-transformed and pooled using the restricted maximum likelihood (REML) random-effects model to account for anticipated between-study clinical heterogeneity [16]. Dichotomous outcomes (e.g. relapse rates) were pooled as risk ratios (RRs) or odds ratios (ORs) using the DerSimonian-Laird random-effects model [17].

*Heterogeneity and subgroup analysis*: Statistical heterogeneity was quantified using Cochran’s Q test (with p<0.10 indicating significance), the I2 statistic (where values >50% represent substantial heterogeneity), and the τ2 statistic [18]. To explore the sources of heterogeneity, predefined subgroup analyses and univariate meta-regression were conducted based on leukemia subtype (ALL vs. AML), patient age (pediatric vs. adult), MRD assessment timing (e.g. post-induction vs. pre-transplant), and MRD positivity thresholds (e.g. 0.1% vs. 0.01%). Leave-one-out sensitivity analyses were performed to evaluate the robustness of pooled estimates.

Publication Bias and Certainty of Evidence

Publication bias and small-study effects were visually evaluated using contour-enhanced funnel plot. Statistical asymmetry was tested using Egger’s linear regression [19] and Begg’s rank correlation tests [20] when 10 or more studies were available for a specific outcome. The trim-and-fill method was applied to adjust the pooled estimates if publication bias was detected. The overall certainty of the evidence for both diagnostic and prognostic outcomes was graded independently by two reviewers using the Grading of Recommendations Assessment, Development, and Evaluation (GRADE) framework, which was adapted for diagnostic test accuracy [21]. Disagreements were resolved by adjudication by a third reviewer.

Results

Literature Search and Study Selection

**Figure 1:**
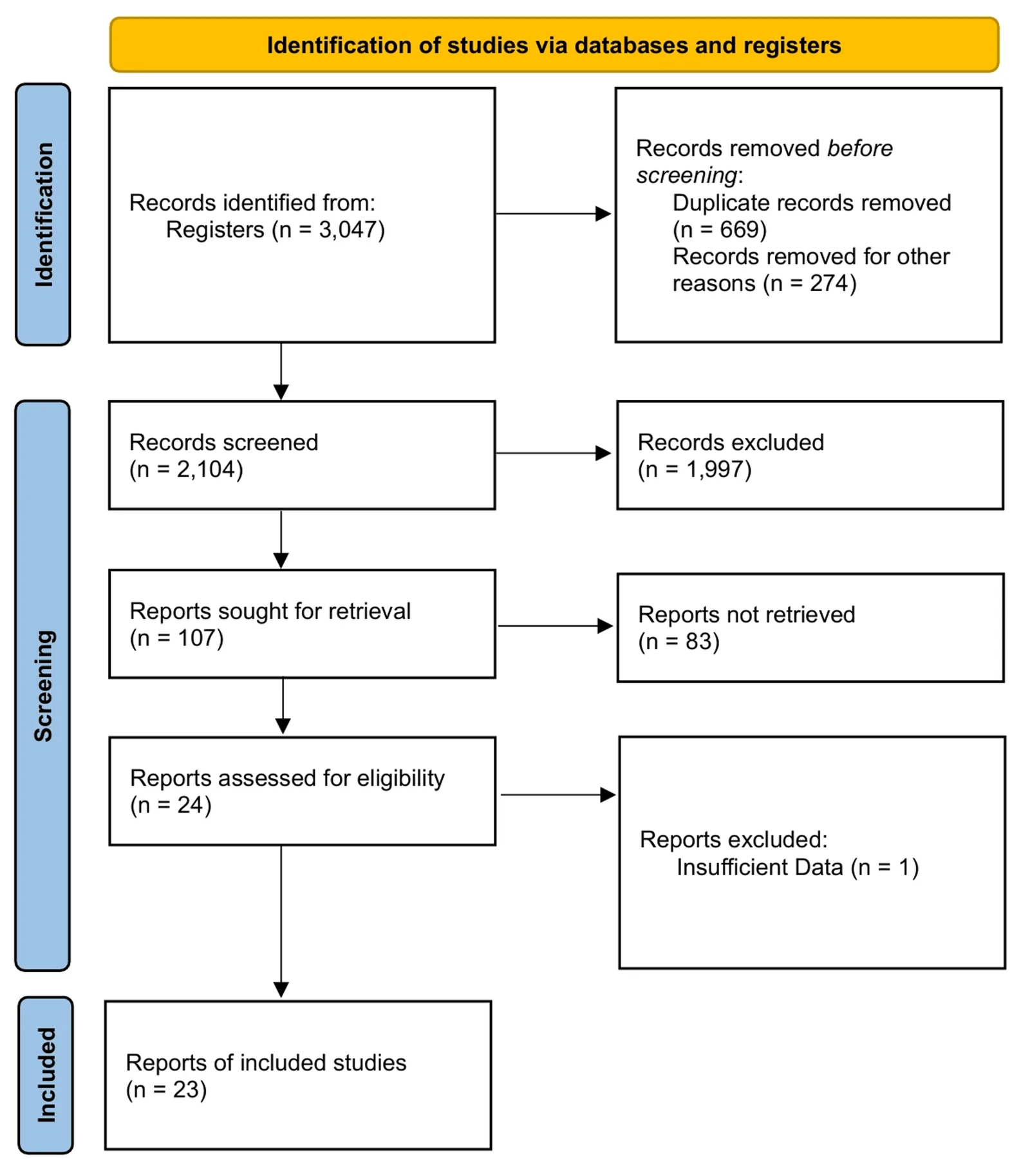
PRISMA 2020 flow diagram. PRISMA: Preferred Reporting Items for Systematic Reviews and Meta-Analyses.

The initial database and registry search yielded 3,047 records screened. After removing 669 duplicates and 274 records for other automated reasons, 2,104 unique titles and abstracts were screened. Of these, 1,997 records were excluded for not meeting the predefined eligibility criteria. Full-text retrieval was sought for 107 reports; 83 were not retrieved (predominantly because they were conference abstracts lacking full-length peer-reviewed publications with extractable data), leaving 24 full-text articles assessed for eligibility. One report was excluded because of insufficient extractable data. Twenty-three non-randomized studies met all inclusion criteria and were incorporated into the systematic review, with data from 12 studies providing 2×2 contingency data for the DTA meta-analysis and six studies providing effect sizes for the prognostic meta-analysis (Figure 1).

Characteristics of the Included Studies

The 23 included studies encompassed a diverse international cohort of patients with leukemia treated across various clinical settings [6-8,22-41]. The studies spanned pediatric, adult, and infant populations, capturing a broad spectrum of hematologic malignancies, including B-cell precursor acute lymphoblastic leukemia (BCP-ALL), T-cell ALL, and AML with specific genetic aberrations (e.g. NPM1 mutations and core-binding factor (CBF) translocations). Methodologically, the index test MFC utilized four- to eight-color panels with positivity thresholds set between 0.01% and 0.1%. The reference standard (PCR-based methodologies, including RQ-PCR and RT-qPCR) evaluated leukemia-specific fusion gene transcripts (FGT) or clonal Ig/TCR rearrangements, with sensitivity thresholds of 0.001%-0.01%. Although CLL and CML were included in the initial search strategy, all studies that met the inclusion criteria for paired MFC and PCR assessments with extractable quantitative data were restricted to ALL and AML cohorts. The baseline characteristics of the included studies are presented in Table 1.

**Table 1:** Study Characteristics of Eligible Included Studies. FGT=Fusion Gene Transcript; BCP=B-Cell Precursor; LAIP=Leukemia-Associated Immunophenotype. The number of patients/samples extracted in this table represents only the specific subset of cases that possessed paired MFC and PCR assessments with sufficient extractable data for 2x2 contingency tables or survival analysis. Therefore, these numbers may be lower than the total cohort sizes reported in the original source articles.

Study (Year)	Country	Study Design	Population	Leukemia Subtype	Index Test (MFC/FCM) Threshold	Reference Test (PCR) Threshold	N (Patients/Samples)
Hrabovsky et al. (2018) [6]	Czech Republic	Retrospective	Adult	ALL	8-color (0.1%)	RQ-PCR (Ig/TCR/FGT)(0.1%)	56/103
Gaipa et al. (2012) [7]	Europe (AIEOP-BFM)	Prospective	Pediatric	ALL	4-color (0.01%)	RQ-PCR (Ig/TCR) (0.01%)	1547/3565
Malec et al. (2004) [8]	Sweden/Netherlands	Retrospective	Pediatric	ALL	3-/4-color (0.01%)	RQ-PCR (Ig/TCR) (0.01%)	22/93
Denys et al. (2012) [22]	Netherlands/Belgium	Retrospective	Pediatric	BCP-ALL, T-ALL	4-/6-color (0.01%)	RQ-PCR (Ig/TCR) (0.01%)	363/898
Popov et al. (2021) [23]	Russia/Belarus	Retrospective	Infant	KMT2A-r ALL	Multicolor (0.01%)	RT-qPCR (FGT) (0.01%)	123/942
Ramos Elbal et al. (2023) [24]	Spain	Retrospective	Pediatric	AML	MFC (0.1%)	RT-qPCR / FISH (0.1%)	20/297
Inaba et al. (2012) [25]	USA (Multicenter)	Prospective	Pediatric	AML	4-color (0.1%)	RQ-PCR (FGT) (0.1%)	203/1514
Rossi et al. (2012) [26]	Italy	Retrospective	Adult	AML	6-color (0.1%)	RQ-PCR (WT1) (90 copies)	23/46
Karlsson et al. (2022) [27]	Nordic Countries	Retrospective	Pediatric	CBF/MLL AML	MFC (0.1%)	RT-qPCR (FGT) (0.1%)	15/22
Wery et al. (2024) [28]	Belgium	Retrospective	Adult	AML / MDS	MFC (LAIP)	RT-qPCR (WT1/FGT)	192/192
Rocha et al. (2019) [29]	Brazil	Retrospective	Pediatric	ALL	4-color (0.01%)	Standard PCR (Qualitative)	42/68
Jung et al. (2024) [30]	South Korea	Retrospective	Adult	B-ALL	MFC (0.01%)	qPCR (BCR-ABL) (0.01%)	182/646
Chang et al. (2026) [31]	China	Retrospective	Pediatric	Ph+ B-ALL	MFC (LAIP)	RT-qPCR (0.01%)	55/100
Hendricks et al. (2019) [32]	South Africa	Retrospective	Pediatric	B-ALL	MFC (EuroFlow)	qPCR (FGT)	64/64
Shang et al. (2021) [33]	China	Retrospective	Adult/Ped	AML t(8;21)	8-color (0.01%)	RQ-PCR (RUNX1) (0.001%)	124/450
Thörn et al. (2011) [34]	Sweden (Multicenter)	Retrospective	Pediatric	ALL	3-/4-color (0.01%)	RQ-PCR (Ig/TCR) (0.01%)	228/726
Gao et al. (2020) [35]	China	Retrospective	Adult	AML (NPM1)	8-color (0.01%)	RQ-PCR (NPM1) (>0)	93/370
Neale et al. (2004) [36]	USA	Prospective	Pediatric	B-ALL	3-color (0.01%)	RQ-PCR (Ig/TCR) (0.01%)	227/1375
Huang et al. (2017) [37]	Taiwan	Retrospective	Pediatric	B-ALL (Fusion+)	6-color (0.01%)	RQ-PCR (FGT) (0.01%)	55/108
Kerst et al. (2005) [38]	Germany (Multicenter)	Retrospective	Pediatric	ALL	4-color (0.01%)	RQ-PCR (Ig/TCR) (0.01%)	45/105
Ryan et al. (2008) [39]	Ireland	Prospective	Pediatric	ALL	3-color (0.01%)	RQ-PCR (Ig/TCR) (0.01%)	53/151
Theunissen et al. (2017) [40]	Europe (EuroFlow)	Prospective	Pediatric	BCP-ALL	8-color (0.001%)	RQ-PCR (Ig/TCR) (0.001%)	178/377
Alm et al. (2017) [41]	Nordic Countries	Retrospective	Pediatric	ETV6-RUNX1 ALL	FACS (0.1%)	RT-qPCR (0.1%)	29/78

Methodological Quality and Risk of Bias

**Figure 2:**
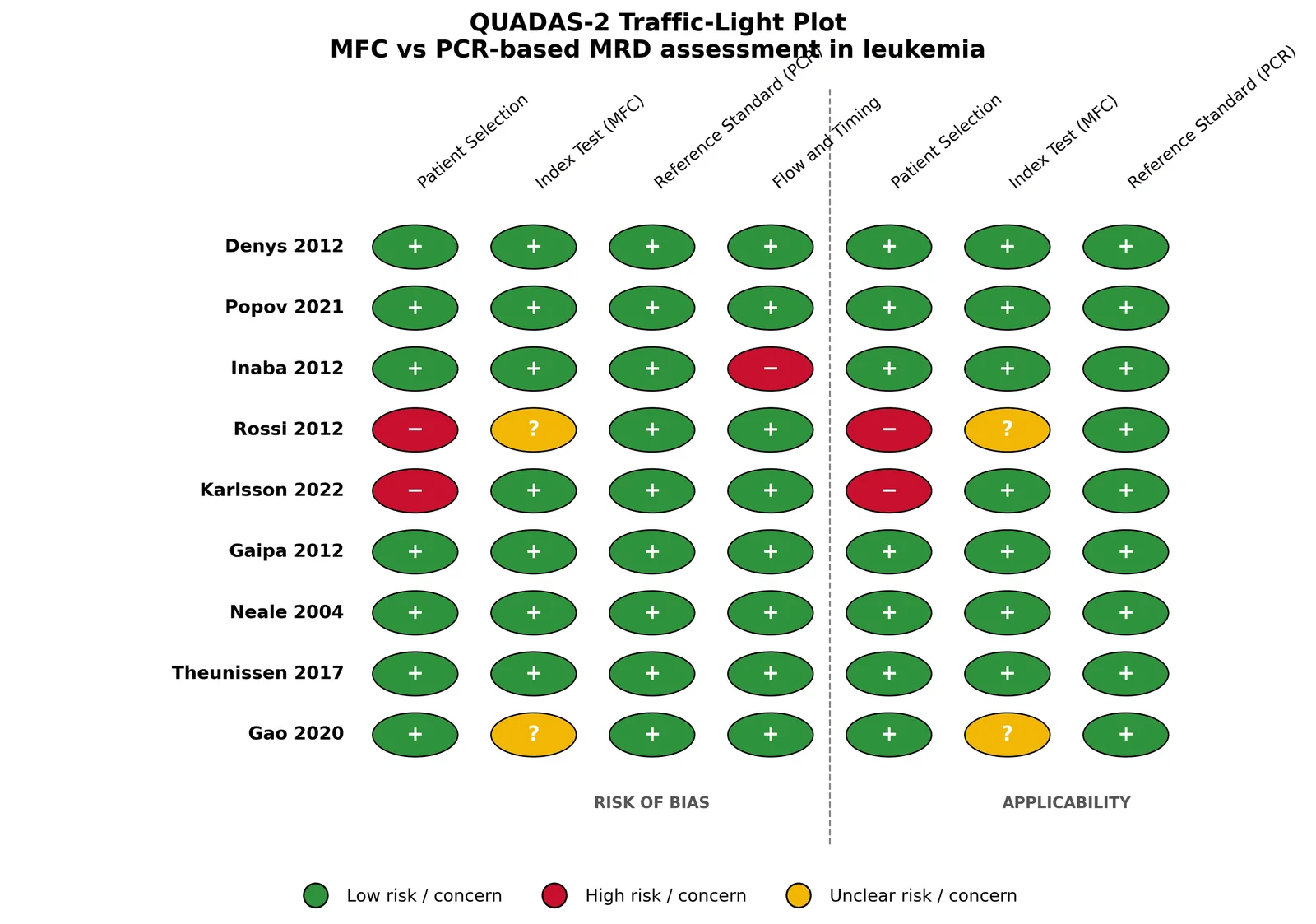
Methodological quality assessment using the QUADAS-2 framework. Traffic-light plot of domain-level judgments for each individual study. The complete aggregate summary of risk of bias for all 12 studies included in the diagnostic accuracy meta-analysis is presented in Figure 3. Studies referenced in the plot: Denys et al. (2012) [22], Popov et al. (2021) [23], Inaba et al. (2012) [25], Rossi et al. (2012) [26], Karlsson et al. (2022) [27], Gaipa et al. (2012) [7], Neale et al. (2004) [36], Theunissen et al. (2017) [40], and Gao et al. (2020) [35].

**Figure 3:**
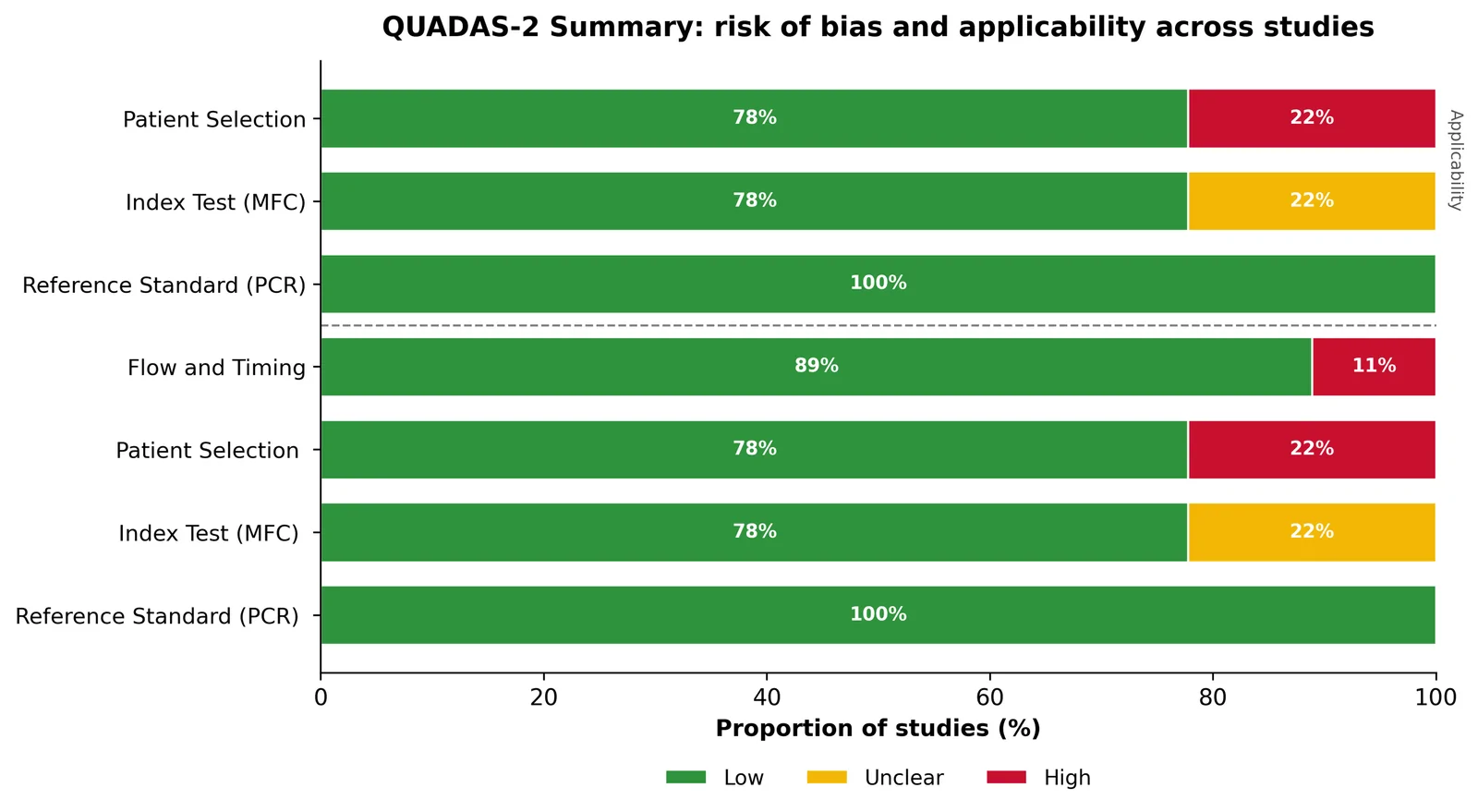
Risk of Bias Summary. Bar plot displays the overall proportion of studies at low, high, or unclear risk of bias and applicability concerns.

Methodological quality for the 12 studies providing quantitative diagnostic test accuracy data was appraised using the QUADAS-2 framework, revealing a robust study design across the included literature (Figures 2,3). As QUADAS-2 is designed for diagnostic studies, the remaining 11 studies providing only prognostic outcomes were not assessed with this tool. The reference standard domain (PCR) was rated as having a low risk of bias (100%). However, a high risk of bias in patient selection was identified in 22% of the studies, driven by the exclusion of specific patient subsets or targeted evaluations (e.g. evaluation of specific translocation subgroups) [26,27]. Furthermore, 22% of studies exhibited an unclear risk of bias regarding the index test, as the blinding of the MFC interpreter to the PCR results was not documented [26,35].

Diagnostic Test Accuracy of MFC versus PCR-Based MRD

**Figure 4:**
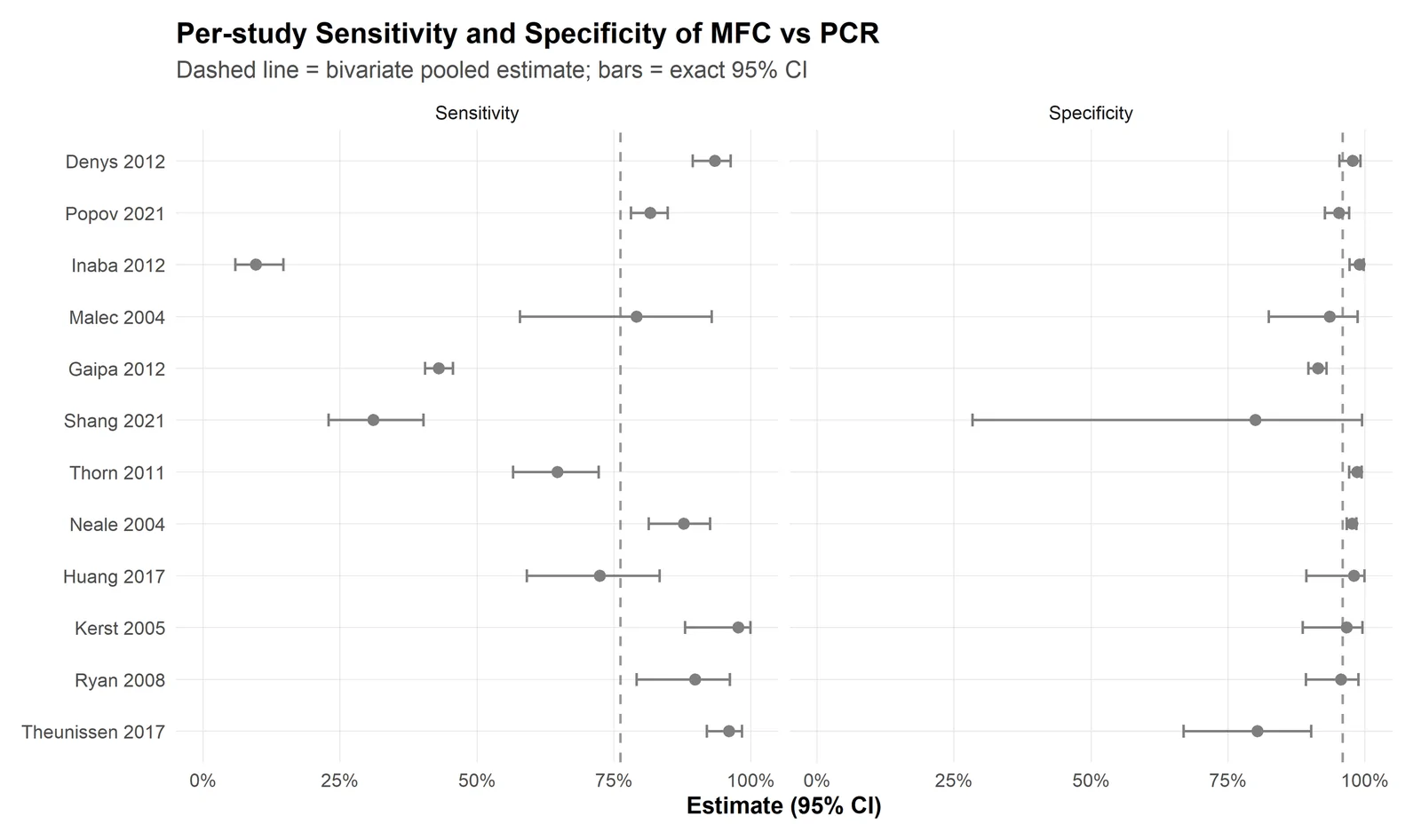
Per-study sensitivity and specificity with exact (Clopper-Pearson) 95% confidence intervals. Coupled forest plots of per-study sensitivity (left) and specificity (right) with exact (Clopper-Pearson) 95% confidence intervals. Dashed lines indicate the bivariate pooled estimates. Studies referenced in the plot: Denys 2012 [22], Popov 2021 [23], Inaba 2012 [25], Malec 2004 [8], Gaipa 2012 [7], Shang 2021 [33], Thörn 2011 [34], Neale 2004 [36], Huang 2017 [37], Kerst 2005 [38], Ryan 2008 [39], and Theunissen 2017 [40].

Data from 12 studies [7,8,22,23,25,33,34,36-40] reporting paired 2×2 contingency tables were synthesized using a bivariate random-effects model, with PCR as the reference standard. MFC demonstrated a moderate pooled sensitivity of 76.2% (95% CI, 55.2-89.3) and an excellent pooled specificity of 96.0% (95% CI, 93.2-97.7) (Table 2; Figure 4). The corresponding false-positive rate was remarkably low at 4.0% (95% CI, 2.3-6.8).

**Table 2:** Pooled diagnostic test accuracy estimates for MFC referenced against PCR-based MRD. *pAUC: Partial area under the curve over the observed range of false-positive rates. DSL: DerSimonian-Laird; HSROC: hierarchical summary receiver operating characteristic.

Diagnostic Metric	Pooled Estimate	95% Confidence Interval	Statistical Model
Sensitivity	0.762 (76.2%)	0.552-0.893	Bivariate (Reitsma) [14]
Specificity	0.960 (96.0%)	0.932-0.977	Bivariate (Reitsma) [14]
Positive Likelihood Ratio (PLR)	15.45	8.00-29.85	Univariate DSL [17]
Negative Likelihood Ratio (NLR)	0.237	0.168-0.334	Univariate DSL [17]
Diagnostic Odds Ratio (DOR)	79.54	25.18-251.24	Univariate DSL [17]
HSROC AUC	0.965	pAUC: 0.87*	HSROC Regression [15]

**Figure 5:**
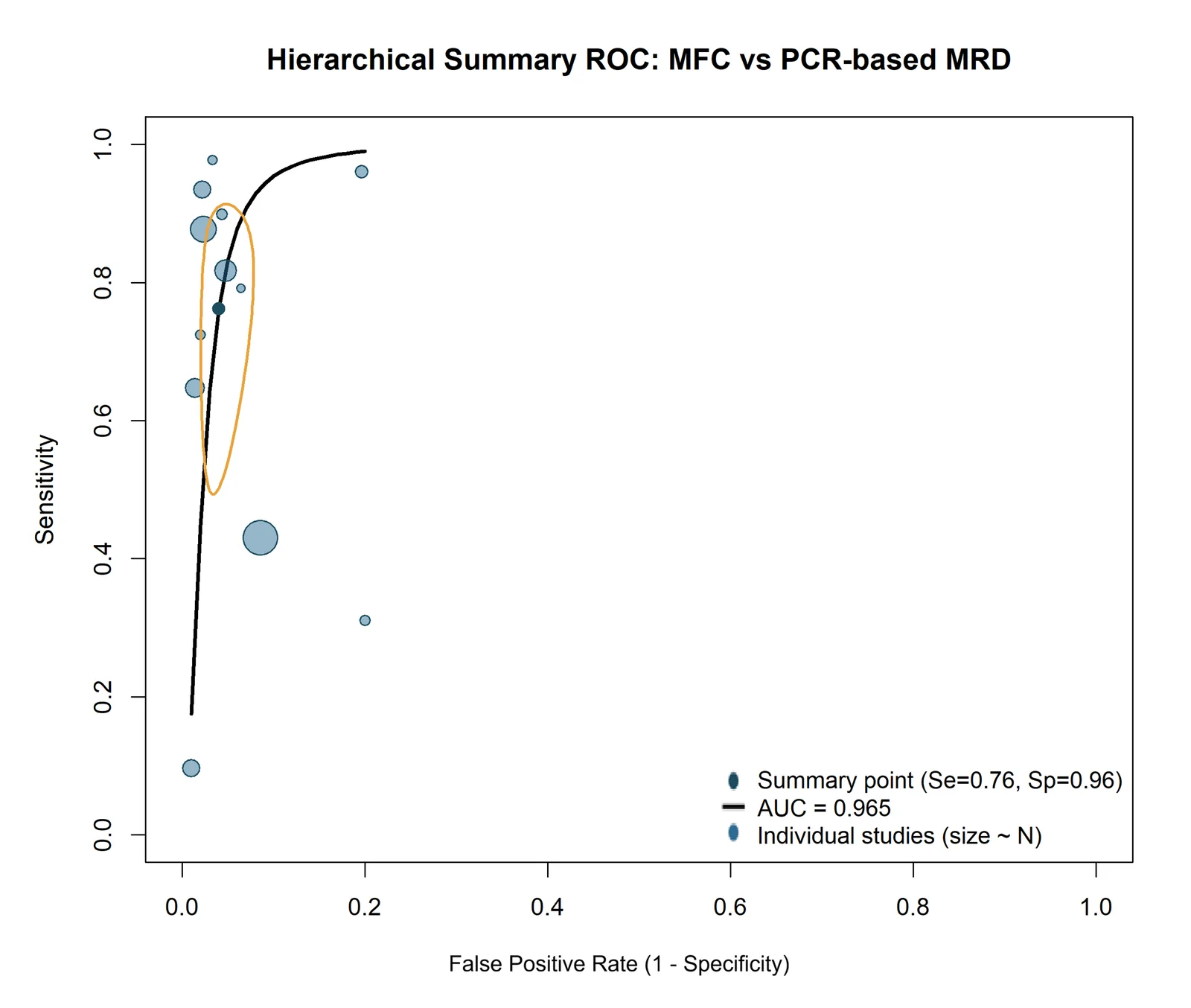
Hierarchical summary receiver operating characteristic (HSROC) curve for MFC versus PCR-based MRD. The dark central point represents the bivariate summary operating point (sensitivity 0.76, specificity 0.96) surrounded by its 95% confidence region; AUC=0.965.

**Figure 6:**
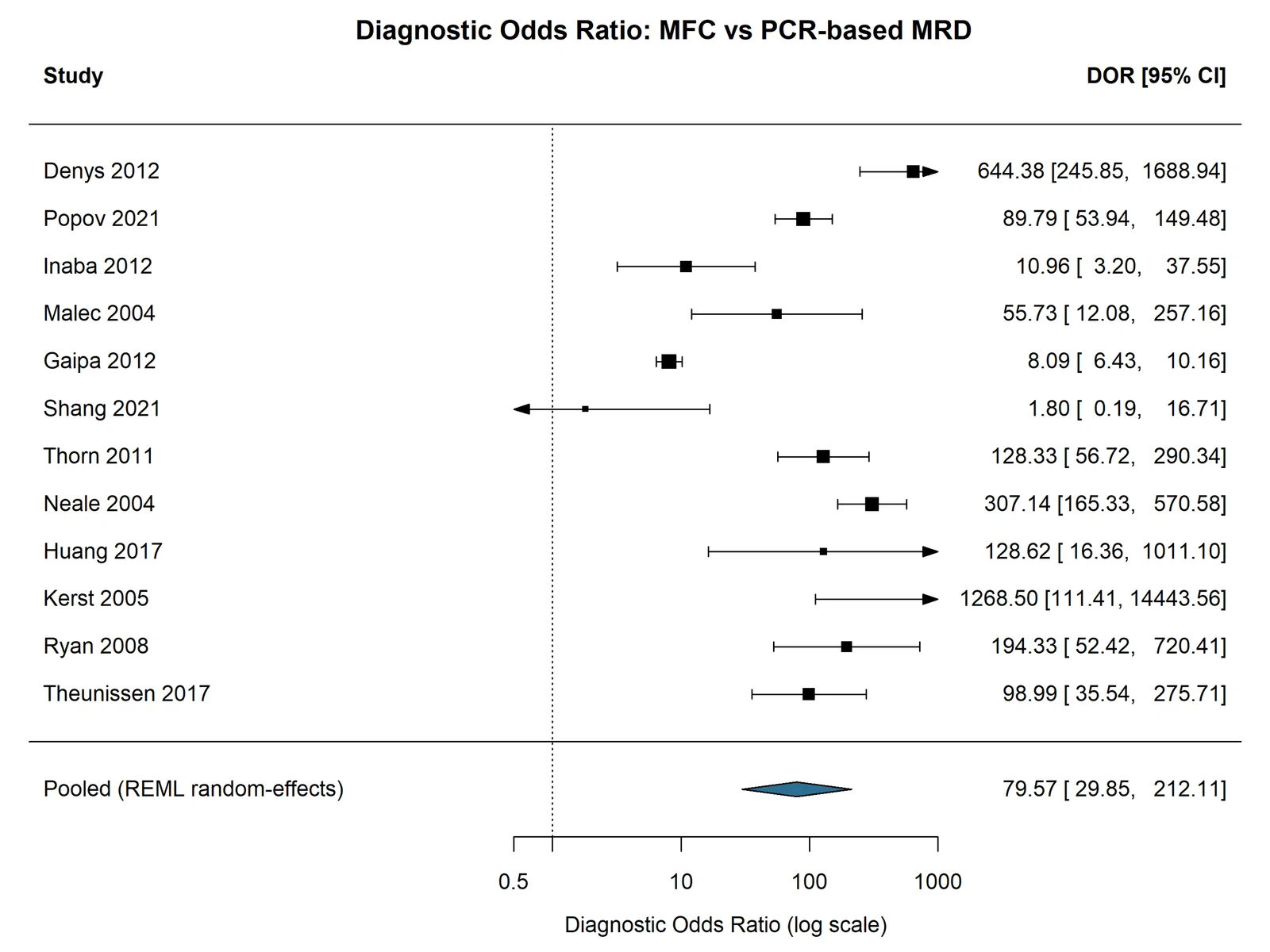
Forest plot of study-level diagnostic odds ratios (DOR) plotted on a logarithmic scale, featuring the REML random-effects pooled estimate. Studies referenced in the plot: Denys et al. (2012) [22], Popov et al. (2021) [23], Inaba et al. (2012) [25], Malec et al. (2004) [8], Gaipa et al. (2012) [7], Shang et al. (2021) [33], Thörn et al. (2011) [34], Neale et al. (2004) [36], Huang et al. (2017) [37], Kerst et al. (2005) [38], Ryan et al. (2008) [39], and Theunissen et al. (2017) [40].

Overall diagnostic discrimination was excellent, characterized by an HSROC curve of 0.965 (Figure 5). To facilitate clinical interpretation, likelihood ratios and DOR were computed using a univariate DerSimonian-Laird approach. MFC yielded a high PLR of 15.45 (95% CI, 8.00-29.85) and a NLR of 0.237 (95% CI, 0.168-0.334). The pooled DOR was 79.54 (95% CI, 25.18-251.24) (Figure 6), indicating that a positive MFC result strongly increases the probability of PCR-detectable residual disease, serving as a highly reliable rule-in test, whereas a negative MFC result provides only moderate rule-out capacity.

Heterogeneity, Subgroup Analyses, and Meta-Regression

Substantial between-study heterogeneity was observed in the log-DOR under the REML random-effects model (Cochran’s Q=276.67, df=11, p<0.0001; I^2^=94.4%; τ^2^=2.55). To elucidate the sources of this heterogeneity, pre-specified univariate meta-regression and subgroup analyses were conducted (Table 3).

**Table 3:** Moderator analyses and univariate meta-regression exploring sources of heterogeneity for the log-DOR. Significant findings (p<0.05) are bolded. AML studies were associated with a significantly lower log-DOR compared to ALL studies.

Moderator / Subgroup	QM​ Test Statistic	P -value	Residual I^2^	R^2^ (Heterogeneity Explained)
Leukemia Subtype (ALL vs. AML)	QM​=7.27	0.0070	91.8%	36.7%
Age Group (Pediatric vs. Adult/Mixed)	QM​=4.39	0.111	92.7%	13.6%
MRD Positivity Threshold (e.g., 0.1% vs 0.01%)	QM​=1.36	0.507	95.2%	0.0%
Study Sample Size (logN)	Slope	0.674	93.5%	—

**Figure 7:**
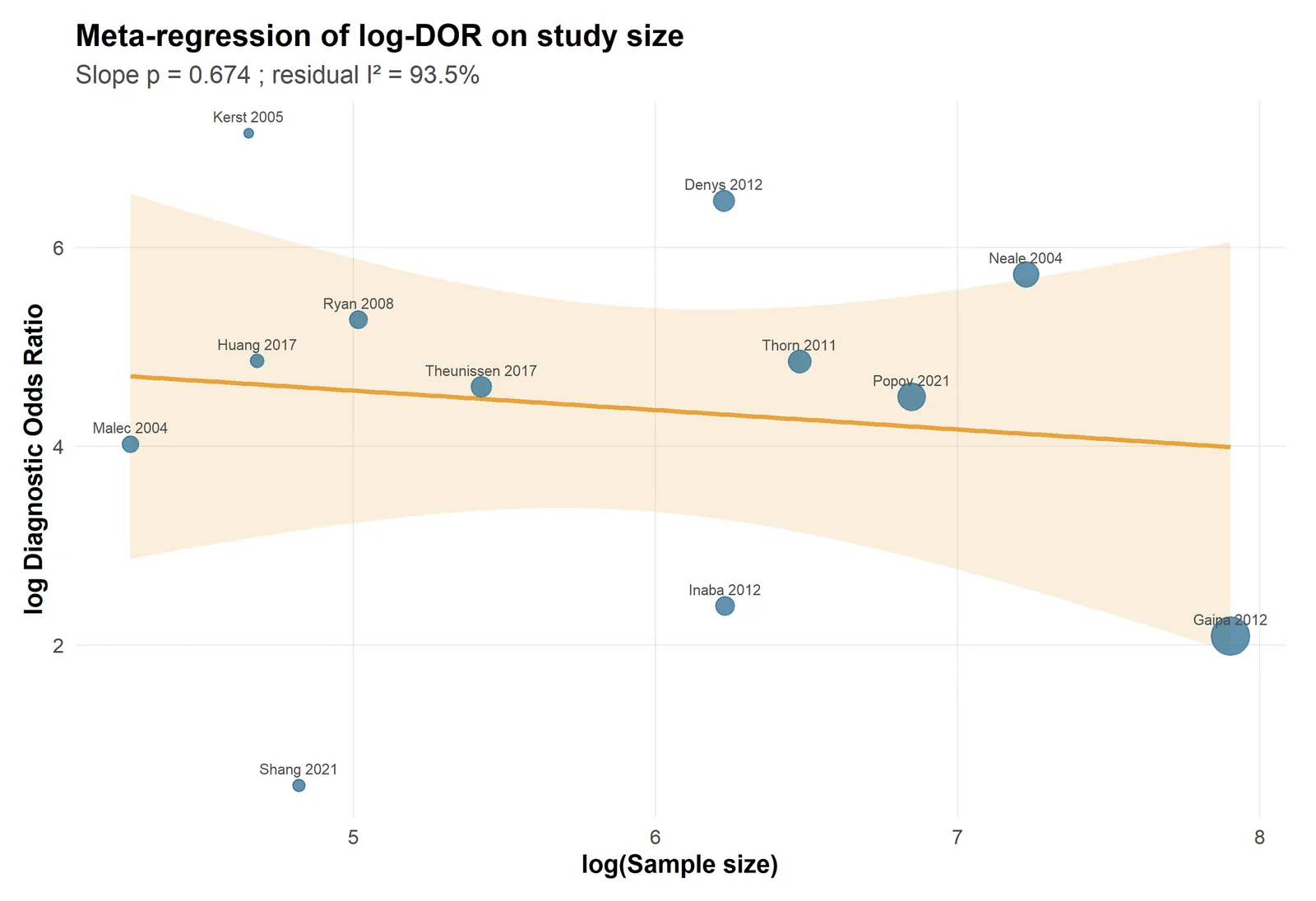
Meta-regression bubble plot of log-DOR against log sample size. Bubble area is proportional to study weight. Studies referenced in the plot: Denys et al. 2012 [22], Popov et al. 2021 [23], Inaba et al. 2012 [25], Malec et al. 2004 [8], Gaipa et al. 2012 [7], Shang et al. 2021 [33], Thörn et al. 2011 [34], Neale et al. 2004 [36], Huang et al. 2017 [37], Kerst et al. 2005 [38], Ryan et al. 2008 [39], and Theunissen et al. 2017 [40].

Leukemia subtype emerged as the sole statistically significant moderator (QM​=7.27, p=0.0070). Specifically, AML studies exhibited a lower log-DOR than ALL studies (coefficient -3.18, 95% CI, -5.48 to -0.87), accounting for 36.7% of the observed heterogeneity. Variables, including age group (p=0.111), MRD threshold (p=0.507), and study sample size (p=0.674), did not significantly influence the diagnostic performance (Figure 7).

Sensitivity Analysis and Publication Bias

**Figure 8:**
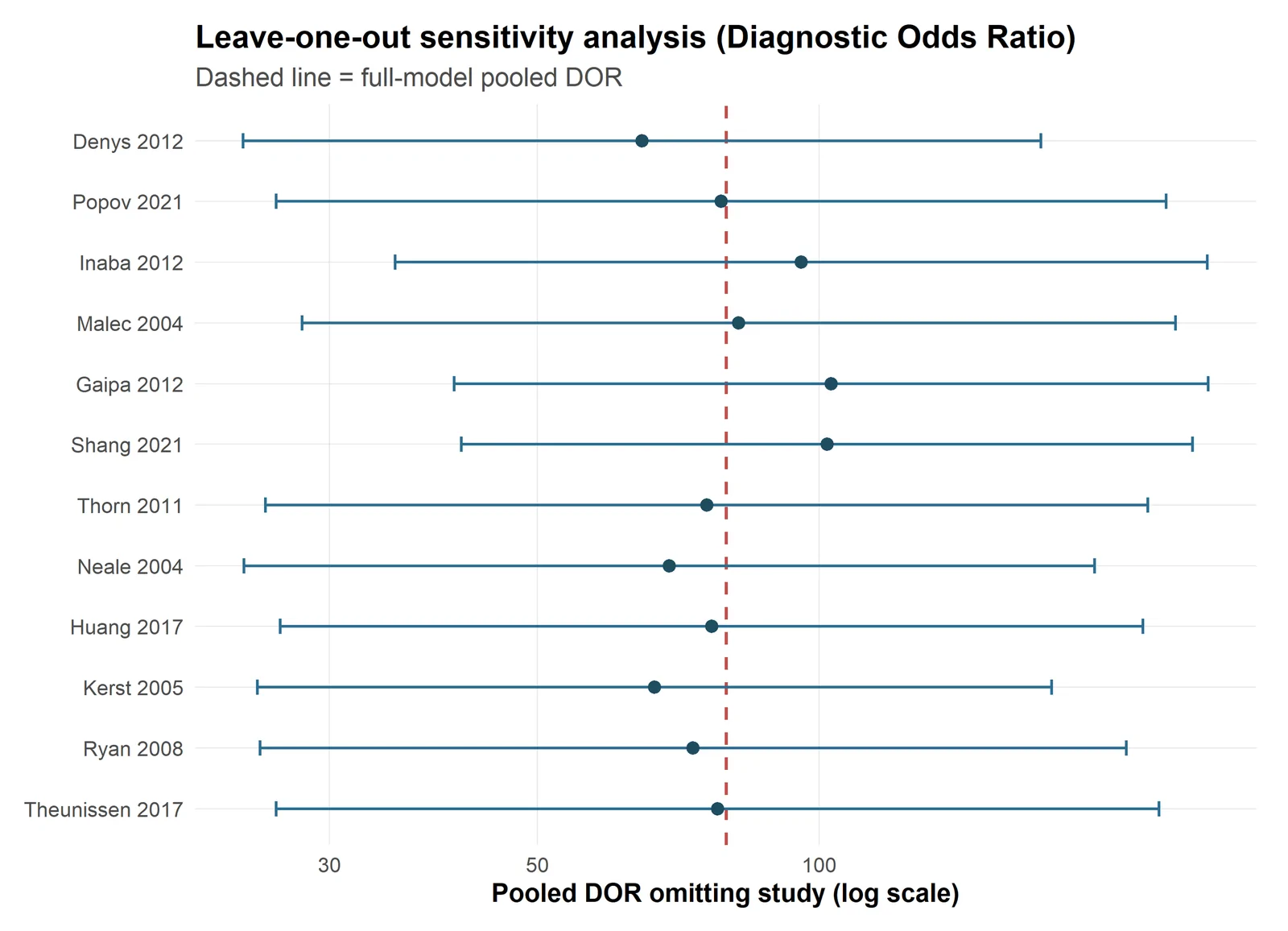
Leave-one-out sensitivity analysis. Plot demonstrates the stability of the pooled diagnostic odds ratio (log scale) upon sequential omission of individual studies. Studies referenced in the plot: Denys et al. 2012 [22], Popov et al. 2021 [23], Inaba et al. 2012 [25], Malec et al. 2004 [8], Gaipa et al. 2012 [7], Shang et al. 2021 [33], Thörn et al. 2011 [34], Neale et al. 2004 [36], Huang et al. 2017 [37], Kerst et al. 2005 [38], Ryan et al. 2008 [39], and Theunissen et al. 2017 [40].

A leave-one-out sensitivity analysis confirmed the robustness of the diagnostic estimates. Sequentially omitting individual studies left the pooled DOR essentially unchanged, fluctuating within a narrow confidence band around the full-model estimate of ~79.5 (Figure 8).

**Figure 9:**
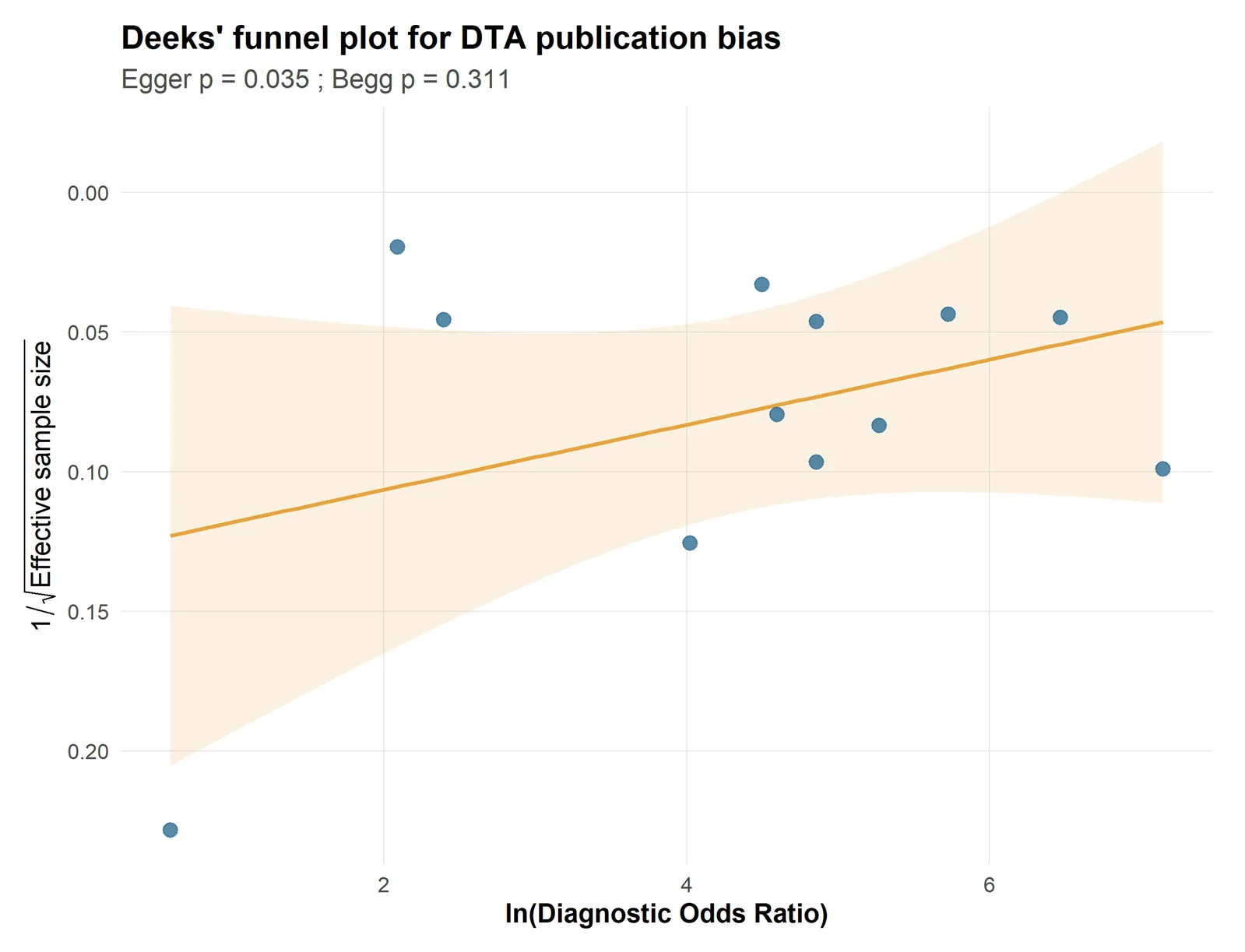
Deeks’ funnel plot for the assessment of diagnostic test accuracy publication bias (inverse root effective sample size vs ln DOR).

The publication bias for diagnostic accuracy was visually assessed using a Deeks’-style funnel plot (Figure 9). Statistical evaluation revealed a significant Egger’s linear regression test (t=2.43, df=10, p=0.035), although Begg’s rank correlation test remained non-significant (Kendall’s τ=-0.24, p=0.311). This mixed signal provides weak-to-moderate evidence of small study effects or publication bias. However, given the subtype-related heterogeneity identified in the meta-regression, these asymmetry tests should be interpreted with caution.

Prognostic Value of MFC/PCR Discordance

**Figure 10:**
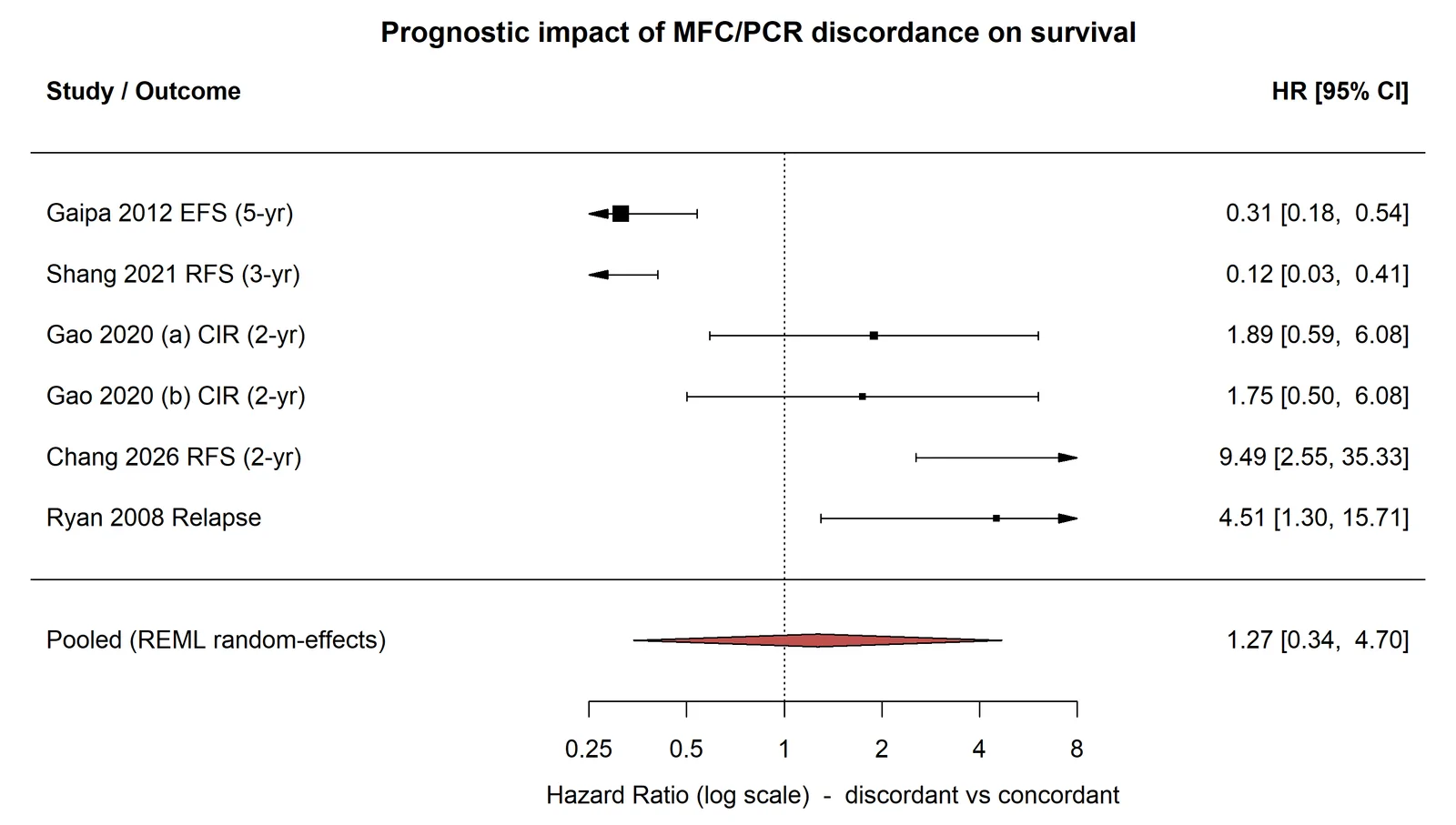
Forest plot of hazard ratios (HR) for survival outcomes based on MFC/PCR discordance. The direction of the effect size is highly dependent on the utilized reference group (double-positive vs. double-negative). Studies referenced: Gaipa et al. 2012 [7], Shang et al. 2021 [33], Gao et al. 2020 [35], Chang et al. 2026 [31], and Ryan et al. 2008 [39]. The notations (a) and (b) corresponding to Gao et al. 2020 [35] are utilized to distinguish between different clinical subgroups or specific analytical thresholds evaluated for survival outcomes within the same primary study.

**Figure 11:**
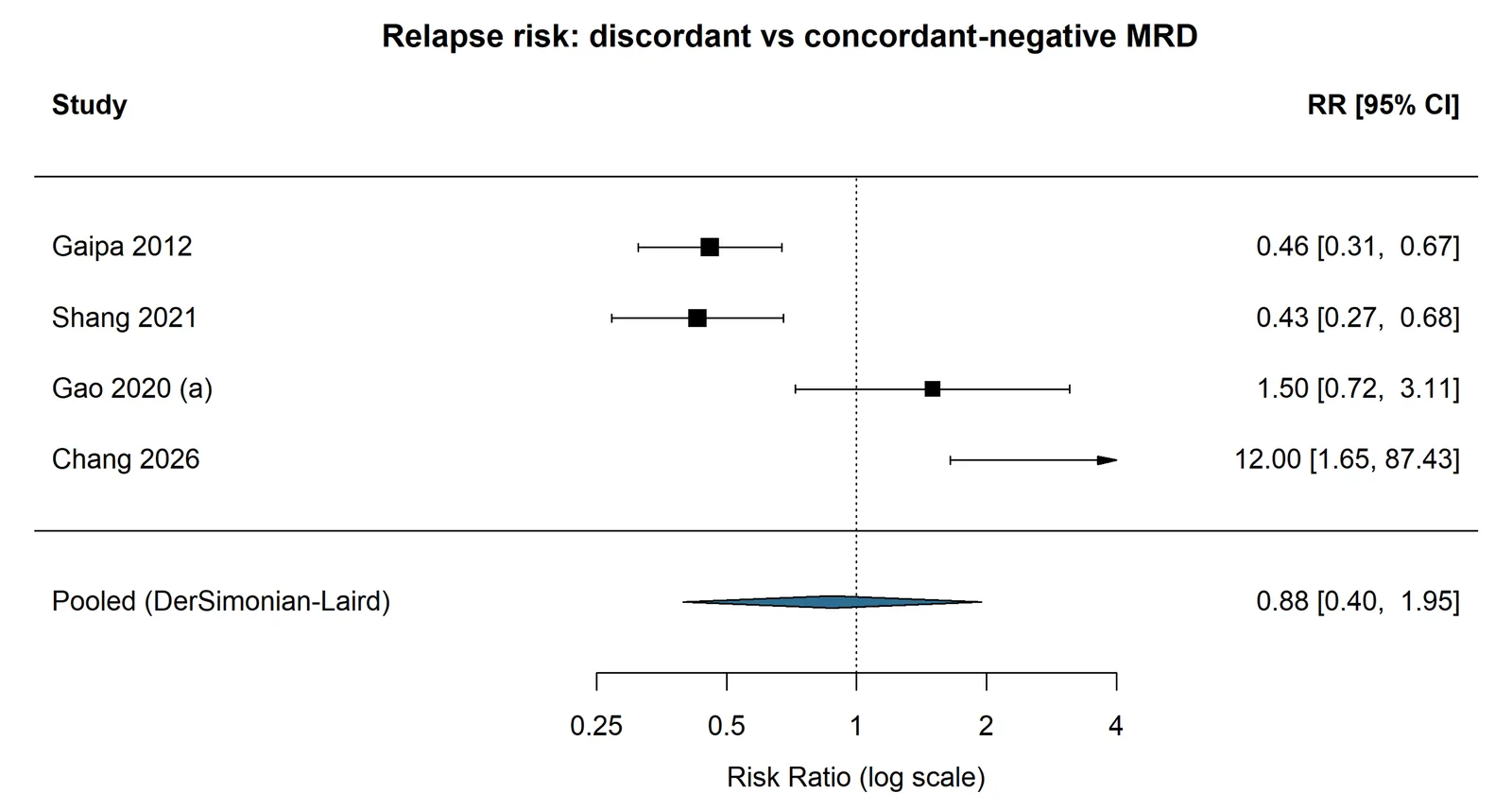
Forest plot of risk ratios (RR) for leukemia relapse contrasting discordant versus concordant-negative MRD states. Studies referenced: Gaipa et al. (2012) [7], Shang et al. (2021) [33], Gao et al. (2020) [35], and Chang et al. (2026) [31]. The notations (a) and (b) corresponding to Gao et al. (2020) [35] are utilized to distinguish between different clinical subgroups or specific analytical thresholds evaluated for survival outcomes within the same primary study.

Six survival comparisons from five studies provided extractable data to contrast the prognostic impact of discordant MFC/PCR states with that of concordant reference groups. Pooled on the log scale utilizing a REML random-effects model, the overall HR for survival based on MFC/PCR discordance was 1.27 (95% CI, 0.34-4.70; p=0.720) (Figure 10). Similarly, the pooled RR for relapse (incorporating four studies) was 0.88 (95% CI, 0.40-1.95) (Figure 11).

**Figure 12:**
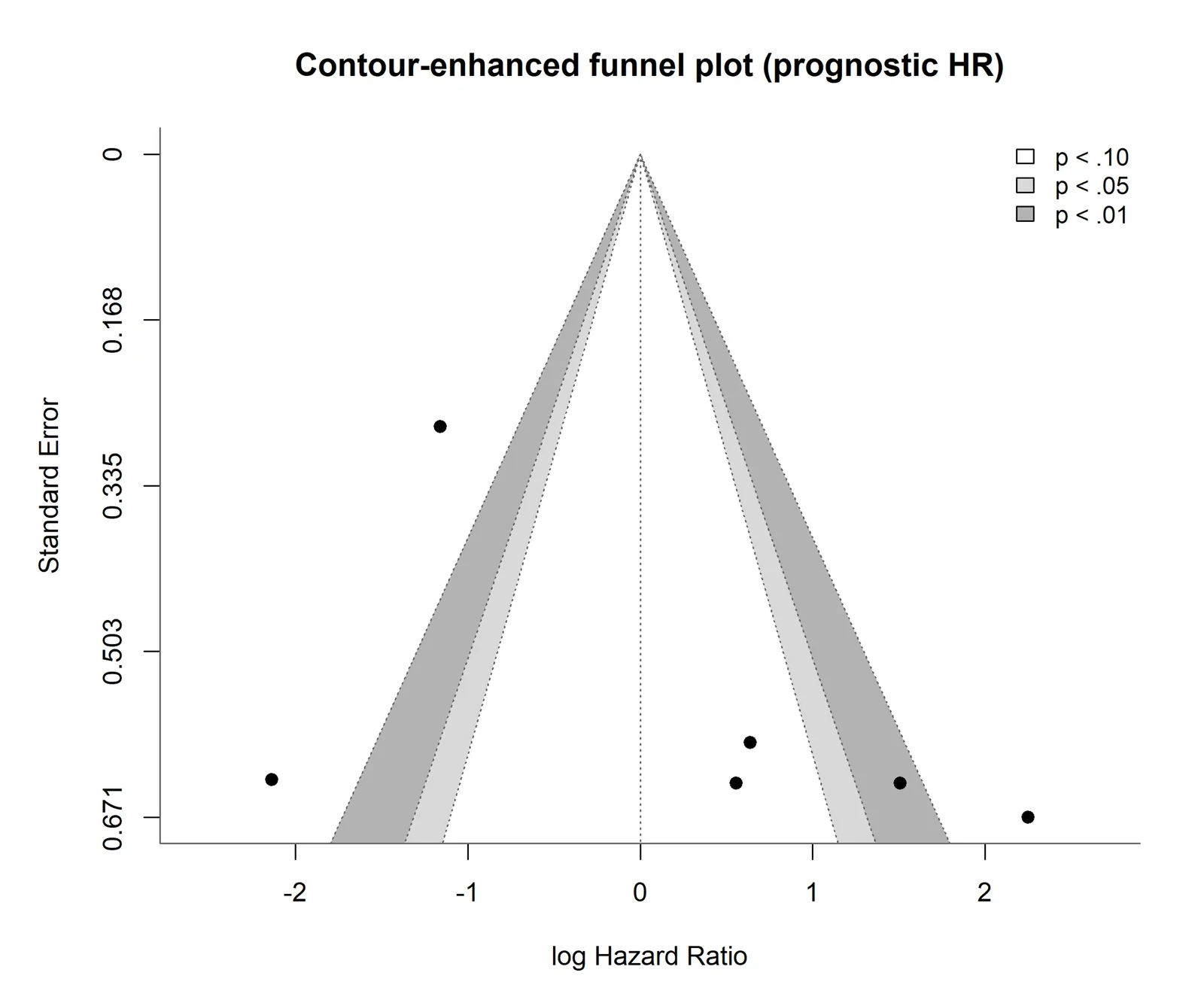
Contour-enhanced funnel plot evaluating small-study effects and publication bias for prognostic hazard ratios.

Both prognostic estimates were statistically non-significant and showed extreme heterogeneity (I2=89.2% for survival; I2=83.8% for relapse risk). This non-significance masks a directional divergence that is dependent on the assigned reference group. Studies contrasting isolated PCR positivity (MFC−/PCR+) against double-positive disease (MFC+/PCR+) found the discordant state to be prognostically favourable (e.g., Gaipa et al. [7] HR 0.31; Shang et al. [33] HR 0.12), while studies contrasting the exact same discordant state against double-negative disease (MFC−/PCR−) found it to carry a substantially higher clinical risk (e.g., Chang et al. [31] HR 9.49; Ryan et al. [39] HR 4.51). Therefore, the prognostic implications of MFC/PCR discordance are not uniform; they are highly context-dependent and influenced by the baseline comparator. Small-study effects for prognostic outcomes were visually mapped, although formal statistical testing was precluded by the limited number of effect sizes (k=6) (Figure 12).

Certainty of Evidence (GRADE)

The certainty of the synthesized evidence was evaluated using the GRADE framework [21], which was adapted for diagnostic tests (Table 4). The certainty for core diagnostic accuracy metrics (Sensitivity, Specificity, AUC) was deemed moderate, downgraded one level due to severe inconsistency (I2>90%). The certainty for the DOR was rated as low, downgraded for both inconsistency and indirectness (arising from the pooling of mixed ALL/AML and pediatric/adult cohorts). The certainty for prognostic outcomes (EFS/RFS and relapse/CIR) was classified as low to very low, penalized for risk of bias (reliance on estimated HRs from survival curves), severe inconsistency, and imprecision driven by sparse event rates across a limited number of studies.

**Table 4:** GRADE Summary of Findings outlining the certainty of evidence for major diagnostic and prognostic outcomes. ^a^ Inconsistency: Downgraded one level due to substantial between-study statistical heterogeneity (I2>90% for DOR; I2=94.4%). ^b^ Indirectness: Downgraded one level due to the pooling of distinct biological states (mixed ALL and AML cohorts), which influenced the test performance, as confirmed by meta-regression. ^c^ Risk of Bias: Downgraded one level because several hazard ratios (HR) were not directly reported and had to be estimated from published Kaplan-Meier survival curves using the Tierney method [11], introducing potential estimation error. ^d^ Inconsistency: Downgraded one level due to extreme heterogeneity (I2>80%) driven by the directional divergence of the effect size, which depended on whether the comparator was double-negative or double-positive disease. ^e^ Imprecision: Downgraded one level due to a small number of studies (k=4) and relatively sparse events in the discordant subpopulations. ^f^ Prognostic evaluations utilizing non-randomized observational data inherently start at low certainty in standard GRADE frameworks; however, because these are derived from objective diagnostic test evaluations, they were evaluated from a high baseline to maintain consistency with the DTA grading methodology. ^g^ Indirectness:  One level downgraded due to heterogeneous definitions of relapse endpoints across studies (e.g. morphological vs. molecular relapse).

Outcome	No. of Studies	Risk of Bias	Inconsistency	Indirectness	Imprecision	Publication Bias	Certainty of Evidence
Sensitivity (MFC vs. PCR) (Diagnostic accuracy)	12 [7,8,22,23,25,33,34,36-40]	Not Serious	Serious (-1)^a^	Not Serious	Not Serious	Not Serious	⊕⊕⊕⊝ MODERATE
Specificity (MFC vs. PCR) (Diagnostic accuracy)	12 [7,8,22,23,25,33,34,36-40]	Not Serious	Serious (-1)^a^	Not Serious	Not Serious	Not Serious	⊕⊕⊕⊝ MODERATE
Diagnostic Odds Ratio (DOR) (Diagnostic accuracy)	12 [7,8,22,23,25,33,34,36-40]	Not Serious	Serious (-1)^a^	Serious (-1)^b^	Not Serious	Not Serious	⊕⊕⊝⊝ LOW
Summary AUC (HSROC) (Diagnostic accuracy)	12 [7,8,22,23,25,33,34,36-40]	Not Serious	Serious (-1)^a^	Not Serious	Not Serious	Not Serious	⊕⊕⊕⊝ MODERATE
EFS/RFS (Discordant vs. Concordant MRD) (Prognostic - survival time)	5 [7,31,33,35,39]	Serious (-1)^c^	Serious (-1)^d^	Not Serious	Serious (-1)e	Not Serious	⊕⊝⊝⊝ LOW^f^
Relapse / CIR (Discordant vs. Concordant MRD) (Prognostic - dichotomous events)	4 [7,31,33,35]	Serious (-1)^c^	Serious (-1)^d^	Serious (-1)^g^	Serious (-1)^e^	Not Serious	⊝⊝⊝⊝ VERY LOW^f^

Discussion

This systematic review and meta-analysis synthesized data from 23 studies to elucidate the comparative diagnostic accuracy and prognostic implications of MFC versus PCR-based MRD assessment in leukemia. The findings confirm that while MFC provides excellent overall discrimination (AUC=0.965) and exceptional specificity (96.0%), its pooled sensitivity remains moderate (76.2%) when compared with PCR. The meta-regression identified a subtype-specific divergence, demonstrating that MFC performs significantly less optimally in AML than in ALL. Furthermore, the prognostic synthesis revealed that inter-method discordance, specifically the MFC−/PCR+ state, does not confer a uniform clinical risk but is highly context-dependent, serving as a critical intermediate-risk state between deep molecular remission and overt macroscopic residual disease.

The high specificity of the MFC observed in our analysis underscores its utility as a reliable rule-in test. A positive MFC result generated a high PLR (15.45), indicating that the flow cytometric detection of aberrant blasts is rarely false-positive when referenced against molecular persistence. The instances of false positivity (MFC+/PCR−, 4.0%) observed in the literature are attributable to the detection of regenerating normal hematopoietic progenitors (e.g. hematogones in B-ALL) that mimic LAIPs or, conversely, the actual loss of PCR molecular targets due to clonal evolution [7,35,40]. The advent of highly standardized next-generation flow cytometry protocols, such as those developed by the EuroFlow Consortium, has mitigated these false-positives by utilizing sophisticated eight-color panels and bulk-lysis procedures to evaluate ≥4×106 cells, pushing MFC sensitivity closer to that of PCR (10-5) [40], complementing international molecular standardization efforts such as the EuroMRD guidelines [42].

However, the moderate pooled sensitivity (76.2%) highlights the limitations of the standard MFC. A negative MFC result cannot rule out the presence of submicroscopic disease, as reflected by the modest NLR (0.237). This limitation was illuminated by our meta-regression, which demonstrated that the DOR for MFC was significantly lower in AML cohorts than in ALL cohorts (p=0.0070). This biological discrepancy is multifactorial in nature. In ALL, PCR targets highly specific, clonally rearranged Ig/TCR genes, whereas MFC targets well-defined B- or T-cell developmental aberrancies [8,38]. AML is characterized by intratumoural heterogeneity, asynchronous maturational arrest, and a high frequency of immunophenotypic shifts following targeted or cytotoxic therapies [24,26,32]. AML LAIPs overlap with dysplastic or regenerating myelopoiesis, which diminishes the sensitivity of flow cytometry [2,26]. For AML, particularly in subtypes defined by distinct molecular aberrations (e.g. NPM1 mutations or RUNX1::RUNX1T1 fusions), PCR remains the indispensable gold standard for deep MRD quantification [33,35].

The central objective of this meta-analysis was to decode the prognostic significance of MFC/PCR discordance, a frequent clinical conundrum. Our pooled survival analysis yielded a non-significant hazard ratio (HR=1.27) with extreme statistical heterogeneity (I2=89.2%), which is not statistical noise; rather, it reflects a vital clinical reality: MFC/PCR discordance is not a biological monolith. When studies compared the isolated PCR-positive state (MFC−/PCR+) against double-positive disease (MFC+/PCR+), the discordant patients exhibited superior survival outcomes [7,33], while when this exact same discordant state was compared against double-negative patients (MFC−/PCR−), it portended a higher risk of relapse [31,39], indicating that the MFC−/PCR+ state represents an intermediate tumor burden, with disease levels low enough to evade MFC detection but high enough to be captured by PCR amplification. Thus, while isolated PCR positivity is clinically preferable to double-positive disease, achieving a dual-negative state (deep molecular remission) remains a prerequisite for long-term cure, particularly prior to allogeneic HSCT [4,28]. However, it is crucial to interpret these findings with caution. The extreme statistical heterogeneity (I²>80%) observed in both diagnostic and prognostic models reflects profound underlying clinical heterogeneity. Because MRD cutoffs, baseline comparators, and patient cohorts varied significantly across studies, our pooled estimates should be viewed as directional trends rather than absolute clinical parameters, thereby reducing the overall certainty of the synthesized evidence.

The clinical implications of these findings advocate for a synergistic rather than competitive application of MRD modalities. MFC is well-suited for early therapeutic time points (e.g. post-induction day 15 or 33) due to its rapid turnaround time, lower cost, and ability to assess treatment kinetics without the need for patient-specific primer design [7,34], whereas PCR should be employed at later milestones (e.g. post-consolidation or pre-transplantation), where disease burden is exponentially lower, and the superior analytical sensitivity of molecular testing is required to guide high-stakes decisions, such as transplant conditioning intensity or the administration of targeted immunotherapies [4,28,35].

This study is strengthened by its rigorous PRISMA-compliant methodology, utilization of hierarchical bivariate models to account for threshold effects, and direct meta-analytical exploration of inter-method discordance on survival. However, the findings of this study must be interpreted in light of several limitations. First, the reliance on predominantly non-randomized, retrospective cohorts introduces selection bias. Second, substantial methodological heterogeneity was present across the included literature, driven by wide variations in MFC gating strategies, antibody panels, PCR techniques, and differing MRD positivity thresholds. This heterogeneity is compounded by the pooling of data across diverse biological environments, most notably pediatric and adult populations. Third, the included studies span a prolonged publication period during which significant technological advancements occurred; pooling older diagnostic technologies (e.g., three- or four-color flow cytometry) with highly standardized contemporary methodologies (e.g., eight-color next-generation flow) contributed to the observed variance and may affect the generalizability of our pooled estimates to modern clinical practice. Fourth, our inability to retrieve a large number of conference abstracts lacking full-text publications may have introduced a degree of publication bias, potentially skewing the synthesis toward studies with statistically significant or favorable outcomes. Finally, while next-generation sequencing (NGS) represents an emerging modality that bridges the high sensitivity of PCR with the broad applicability of MFC, it was not the primary focus of this analysis, though recent data suggest it may eventually supersede standard PCR in clarifying discordant MFC results [30,31].

## Conclusions

MFC- and PCR-based MRD assessments offer distinct and complementary advantages in the management of leukemia. While MFC provides rapid and highly specific prognostic information, PCR remains unparalleled in terms of analytical sensitivity, particularly in AML. Inter-method discordance reflects a spectrum of residual tumor burdens rather than assay failure. Integrating both modalities into subtype-specific clinical algorithms is essential for achieving precise risk stratification, tailoring therapeutic interventions, and improving survival outcomes in patients with leukemia.

## References

[1] Chen X, Wood BL (2022). Minimal residual disease in acute lymphoblastic leukemia: techniques and application. Clinical Management of Acute Lymphoblastic Leukemia.

[2] Haaksma LH, Cloos J, de Leeuw DC (2025). Flow cytometric and molecular MRD in AML: current methods, clinical applications and challenges. Leuk Lymphoma.

[3] Zhang YW, Su L, Tan YH (2023). Measurable residual disease detected by flow cytometry independently predicts prognoses of NPM1-mutated acute myeloid leukemia. Ann Hematol.

[4] Guolo F, Minetto P, Clavio M (2017). Combining flow cytometry and WT1 assessment improves the prognostic value of pre-transplant minimal residual disease in acute myeloid leukemia. Haematologica.

[5] Kaur G, Prakash A, Medhi B (2024). Cytokine analysis in leukemia and minimal residual disease (MRD) detection through flow cytometry. Flow Cytometry.

[6] Hrabovsky S, Folber F, Horacek JM, Stehlikova O, Jelinkova H, Salek C, Doubek M (2018). Comparison of real-time quantitative polymerase chain reaction and eight-color flow cytometry in assessment of minimal residual disease in adult acute lymphoblastic leukemia. Clin Lymphoma Myeloma Leuk.

[7] Gaipa G, Cazzaniga G, Valsecchi MG (2012). Time point-dependent concordance of flow cytometry and real-time quantitative polymerase chain reaction for minimal residual disease detection in childhood acute lymphoblastic leukemia. Haematologica.

[8] Malec M, van der Velden VH, Björklund E (2004). Analysis of minimal residual disease in childhood acute lymphoblastic leukemia: comparison between RQ-PCR analysis of Ig/TcR gene rearrangements and multicolor flow cytometric immunophenotyping. Leukemia.

[9] Page MJ, McKenzie JE, Bossuyt PM (2021). The PRISMA 2020 statement: an updated guideline for reporting systematic reviews. BMJ.

[10] McHugh ML (2012). Interrater reliability: the kappa statistic. Biochem Med (Zagreb).

[11] Tierney JF, Stewart LA, Ghersi D, Burdett S, Sydes MR (2007). Practical methods for incorporating summary time-to-event data into meta-analysis. Trials.

[12] Whiting PF, Rutjes AW, Westwood ME (2011). QUADAS-2: a revised tool for the quality assessment of diagnostic accuracy studies. Ann Intern Med.

[13] (2026). R Core Team. R: A language and environment for statistical computing. https://www.R-project.org/.

[14] Reitsma JB, Glas AS, Rutjes AW, Scholten RJ, Bossuyt PM, Zwinderman AH (2005). Bivariate analysis of sensitivity and specificity produces informative summary measures in diagnostic reviews. J Clin Epidemiol.

[15] Rutter CM, Gatsonis CA (2001). A hierarchical regression approach to meta-analysis of diagnostic test accuracy evaluations. Stat Med.

[16] Viechtbauer W (2010). Conducting meta-analyses in R with the metafor package. J Stat Softw.

[17] DerSimonian R, Laird N (1986). Meta-analysis in clinical trials. Control Clin Trials.

[18] Higgins JP, Thompson SG, Deeks JJ, Altman DG (2003). Measuring inconsistency in meta-analyses. BMJ.

[19] Egger M, Davey Smith G, Schneider M, Minder C (1997). Bias in meta-analysis detected by a simple, graphical test. BMJ.

[20] Begg CB, Mazumdar M (1994). Operating characteristics of a rank correlation test for publication bias. Biometrics.

[21] Schünemann HJ, Oxman AD, Brozek J (2008). Grading quality of evidence and strength of recommendations for diagnostic tests and strategies. BMJ.

[22] Denys B, van der Sluijs-Gelling AJ, Homburg C (2013). Improved flow cytometric detection of minimal residual disease in childhood acute lymphoblastic leukemia. Leukemia.

[23] Popov A, Tsaur G, Verzhbitskaya T (2023). Comparison of minimal residual disease measurement by multicolour flow cytometry and PCR for fusion gene transcripts in infants with acute lymphoblastic leukaemia with KMT2A gene rearrangements. Br J Haematol.

[24] Ramos Elbal E, Fuster JL, Campillo JA (2023). Measurable residual disease study through three different methods can anticipate relapse and guide pre-emptive therapy in childhood acute myeloid leukemia. Clin Transl Oncol.

[25] Inaba H, Coustan-Smith E, Cao X (2012). Comparative analysis of different approaches to measure treatment response in acute myeloid leukemia. J Clin Oncol.

[26] Rossi G, Minervini MM, Carella AM (2012). Comparison between multiparameter flow cytometry and WT1-RNA quantification in monitoring minimal residual disease in acute myeloid leukemia without specific molecular targets. Leuk Res.

[27] Karlsson L, Nyvold CG, Soboli A (2022). Fusion transcript analysis reveals slower response kinetics than multiparameter flow cytometry in childhood acute myeloid leukaemia. Int J Lab Hematol.

[28] Wery AR, Salaroli A, Andreozzi F (2024). Measurable residual disease assessment prior to allogeneic hematopoietic stem cell transplantation in acute myeloid leukemia and myelodysplastic syndromes: a 20-year monocentric study. Ann Hematol.

[29] Rocha JM, Xavier SG, Souza ME, Murao M, de Oliveira BM (2019). Comparison between flow cytometry and standard PCR in the evaluation of MRD in children with acute lymphoblastic leukemia treated with the GBTLI LLA - 2009 protocol. Pediatr Hematol Oncol.

[30] Jung J, Yoon JH, Lee S (2024). Measurable residual disease in adult B lymphoblastic leukemia: A study of concordance between multiparametric flow cytometry. Next-Generation Sequencing of Immunoglobulin Gene Rearrangements, and Quantitative PCR [Abstract]. Blood.

[31] Chang L, Chang J, Zhao B (2026). Immunoglobulin NGS enhance residual disease detection and prognosis in pediatric Ph+ acute lymphoblastic leukemia. Front Immunol.

[32] Hendricks CL, Buldeo S, Pillay D (2019). Comparing morphology, flow cytometry and molecular genetics in the assessment of minimal residual disease in children with B-acute lymphoblastic leukaemia (B-ALL). S Afr J Oncol.

[33] Shang L, Cai X, Sun W, Cheng Q, Mi Y (2022). Time point-dependent concordance and prognostic significance of flow cytometry and real time quantitative PCR for measurable/minimal residual disease detection in acute myeloid leukemia with t(8;21)(q22;q22.1). Cytometry B Clin Cytom.

[34] Thörn I, Forestier E, Botling J (2011). Minimal residual disease assessment in childhood acute lymphoblastic leukaemia: a Swedish multi-centre study comparing real-time polymerase chain reaction and multicolour flow cytometry. Br J Haematol.

[35] Gao MG, Ruan GR, Chang YJ (2020). The predictive value of minimal residual disease when facing the inconsistent results detected by real-time quantitative PCR and flow cytometry in NPM1-mutated acute myeloid leukemia. Ann Hematol.

[36] Neale GA, Coustan-Smith E, Stow P, Pan Q, Chen X, Pui CH, Campana D (2004). Comparative analysis of flow cytometry and polymerase chain reaction for the detection of minimal residual disease in childhood acute lymphoblastic leukemia. Leukemia.

[37] Huang YJ, Coustan-Smith E, Kao HW (2017). Concordance of two approaches in monitoring of minimal residual disease in B-precursor acute lymphoblastic leukemia: Fusion transcripts and leukemia-associated immunophenotypes. J Formos Med Assoc.

[38] Kerst G, Kreyenberg H, Roth C (2005). Concurrent detection of minimal residual disease (MRD) in childhood acute lymphoblastic leukaemia by flow cytometry and real-time PCR. Br J Haematol.

[39] Ryan J, Quinn F, Meunier A (2009). Minimal residual disease detection in childhood acute lymphoblastic leukaemia patients at multiple time-points reveals high levels of concordance between molecular and immunophenotypic approaches. Br J Haematol.

[40] Theunissen P, Mejstrikova E, Sedek L (2017). Standardized flow cytometry for highly sensitive MRD measurements in B-cell acute lymphoblastic leukemia. Blood.

[41] Alm SJ, Engvall C, Asp J, Palmqvist L, Abrahamsson J, Fogelstrand L (2017). Minimal residual disease monitoring in childhood B lymphoblastic leukemia with t(12;21)(p13;q22); ETV6-RUNX1: concordant results using quantitation of fusion transcript and flow cytometry. Int J Lab Hematol.

[42] van der Velden VH, Cazzaniga G, Schrauder A (2007). Analysis of minimal residual disease by Ig/TCR gene rearrangements: guidelines for interpretation of real-time quantitative PCR data. Leukemia.

